# The use of subtotal petrosectomy in cochlear implant candidates with chronic otitis media

**DOI:** 10.1007/s00405-015-3573-1

**Published:** 2015-02-24

**Authors:** Marcin Szymański, Andre Ataide, Thomas Linder

**Affiliations:** 1 Department of Otolaryngology, Head and Neck Surgery, Medical University of Lublin, Lublin, Poland; 2 Department of Otolaryngology, Pequeno Principe Children’s Hospital, Curitiba, Brazil; 3 Department of Otorhinolaryngology, Head and Neck Surgery, Luzerner Kantonsspital, Lucerne, Switzerland

**Keywords:** Subtotal petrosectomy, Cochlear implants, Chronic otitis media, Cholesteatoma, Complications, Imaging

## Abstract

Cochlear implants (CI) candidates with chronic otitis media require special attention and management. The need of opening of the inner ear creates potential routes of spread of infection to subarachnoid spaces and lead to meningitis. The aim of the study was to analyse the technique and complications of subtotal petrosectomy (SP) in cochlear implant candidates with chronic otitis media at three different CI centres. A retrospective study was carried out in three Territory Referral Hospitals. The centres follow Fisch’s philosophy and surgical techniques of SP. The study group consisted of 19 patients, 4 men and 15 women, aged 12–82 years. All patients underwent SP with either primary or staged CI implantation. Indications for single or a staged management, difficulties during surgery and complications were analysed. Skin and muscle flap design in primary and revision cases as well as imaging follow-up strategy are discussed. In 14 patients implantation was performed in a single stage and in 5 cases in two stages. Follow-up ranged from 8 months to 10 years. All the patients use their implants and there were no major nor minor complications. The use of subtotal petrosectomy with cochlear implants is a safe and efficient technique when strict surgical steps and rules are applied. Closure of the external ear canal after previous meatoplasty can be challenging and extreme care dissecting the skin flaps is required. In patients with extensive cholesteatoma, active discharge from the ear with resistant bacteria or an “unstable” situation, the procedure can be staged.

**Electronic supplementary material:**

The online version of this article (doi:10.1007/s00405-015-3573-1) contains supplementary material, which is available to authorized users.

## Introduction

Chronic otitis media or its treatment may cause profound hearing loss or deafness and these postlingually deafened patients may benefit from cochlear implants (CI). Cochlear implants candidates with chronic otitis media require special attention and management. The need of opening of the inner ear through a cochleostomy or via the round window membrane creates potential routes of spread of infection to subarachnoid spaces. Insertion of an electrode in a potentially infected area carries the risks of meningitis and recurrent skin infections over the implant due to biofilm formation [1–4]. Surgery itself may become difficult due to a chronically infected haemorrhagic mucosa in a poorly pneumatised temporal bone and multiple previous surgeries. Therefore, those patients should require a comprehensive planning of their cochlear implant procedure.

Eradication of the disease, avoidance of recurrence, prevention of meningitis and secure placement of the cochlear implant electrode are the aims of surgery. Those aims can be achieved with the use of one single technique: the subtotal petrosectomy (SP). The technique of subtotal petrosectomy was described by Fisch and Mattox [5] over 30 years ago, but was widely accepted by the cochlear implant community only recently [6]. It involves eradication of all accessible pneumatic spaces in the temporal bone, removal of the middle ear mucous membrane, tympanic membrane, skin of the external ear canal with closure of Eustachian tube and external ear canal. SP is a surgical step in infratemporal approaches to remove skull base pathology, but also serves as a treatment of chronic otitis media or temporal bone fractures. Bendet et al. [7] and Issing et al. [8] were the first ones describing groups of patients where SP was used in patients requiring cochlear implantation.

Like every surgical procedure SP also carries a risk of complications, furthermore there can be some objections raised because of the risks of leaving squamous epithelium or active mucosa in an obliterated cavity.

The aim of the study was to analyse the technique and complications of subtotal petrosectomy in cochlear implant candidates with chronic otitis media at three different CI centres.

## Materials and methods

A retrospective study was carried out in 3 Territory Referral Hospitals. The centres follow Fisch’s philosophy and surgical techniques of subtotal petrosectomy. All patients were included in this study who qualified for cochlear implant surgery and had a history and current signs of chronic otitis media. All patients underwent an SP with either primary or staged CI implantation. The study group consisted of 19 patients, 4 men and 15 women, with an age range of 12–82 years (mean 54 years).

All the patients were operated by surgeons who are involved in teaching the Fisch techniques of subtotal petrosectomy at skull base courses and follow the same strict concept and using the same technical equipment. In 16 patients different types of Nucleus Cochlear (Australia) implants and in 3 patients Medel (Austria) devices were used.

Indications for single or a staged management, difficulties during surgery and complications were analysed retrospectively. Complications were classified according to Cohen and Hofmann [9].

The study was approved by the institutional ethical committee.

### Surgical technique

**Fig. 1:**
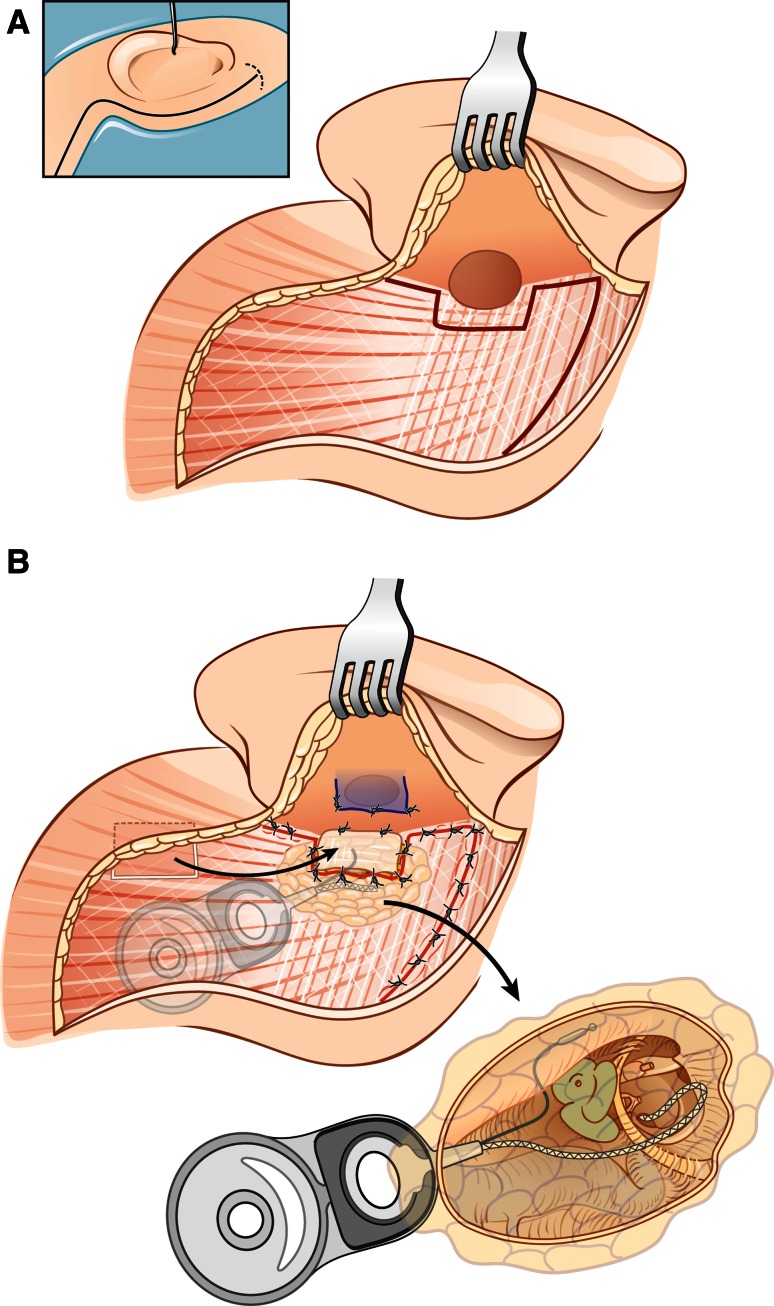
**a** L-shaped retroauricular skin incision and muscle-periosteal flap based posterosuperiorly used in primary cases**. b** Wound closure in primary cases. Cavity is obliterated with abdominal fat. Resuturing of the muscle-periosteal flap with additional free fascia graft from the temporalis muscle

**Fig. 2:**
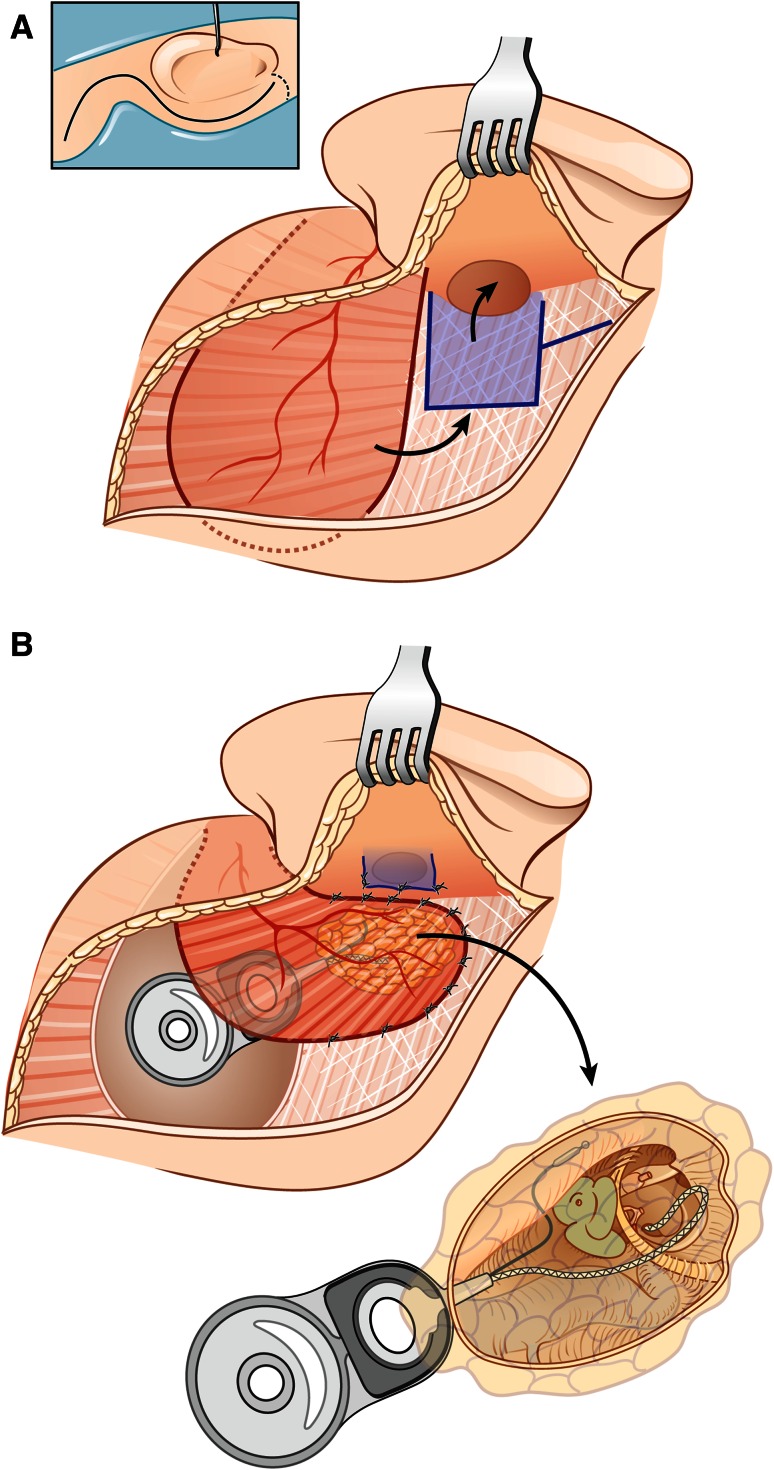
**a** A retroauricular S-shaped incision extending over the temporalis muscle and anteriorly based periosteal flap used in cases operated before**. b** Wound closure in revision cases. The cavity is obliterated using the temporalis muscle rotated inferiorly

There are two main entities regarding the skin incision and soft tissue handling, whereas the bony work remains the same for both approaches:

(A) In case of primary surgery and preserved mastoid periosteum, an L-shaped incision may be used.
(B) In case of revision surgery or in patients with inadequate mastoid periosteal soft tissue remnants a retroauricular S-shape incision with an extended temporalis flap is preferred.

(A) In the infrequent situations, where the ear was never operated before and the retroauricular soft tissues are untouched, an L-shaped retroauricular skin incision was performed and a skin-subcutaneous first flap was raised posteriorly. Next, a rather small periosteal flap was cut with its base anteriorly towards the external ear canal skin. The periosteal incision was extended towards the temporal line superiorly and the mastoid tip inferiorly, coursing backwards over the mastoid tip towards the occipital muscles. This second muscle–periosteal flap was raised posterosuperiorly and is reverse pedicled to the first skin–subcutaneous flap. The external ear canal was transected as described below. At the end, resuturing the muscle–periosteal flap will not allow complete closure at the level of the ear canal (due to the periosteal flap design), but the gap could be filled with a free fascia graft from the temporalis muscle covering the underlying fat (Fig. 1a, b).

(B) In the more frequent situation that the ear had been operated before due to chronic otitis media and the soft tissues over the mastoid have been used for partial obliteration of open or closed cavities, the L-shape approach cannot be advised, since not enough soft tissue is available for the proper wound closure. In all these instances, a retroauricular S-shaped incision was performed extending over the temporalis muscle (Fig. 2a, b). Next the anteriorly based periosteal flap was raised leaving enough soft tissue attachments anteriorly. The posterior ear canal skin was transected 1 mm below the bony entrance and the inferior edge of the tragal cartilage was exposed. A curved clamp was inserted between the parotid gland and the tragal cartilage to protect the frontal branch of the facial nerve and the anterior skin and tragal cartilage were transected at the same level as the posterior skin. One centimetre of skin sleeve was raised separating the skin layer from the tragal and conchal cartilages, everted through the opening of the external ear canal and closed in a first layer using 3–4 resorbable sutures. The second layer was formed by the periosteal flap rotated anteriorly and sutured to the tragal cartilage. This resulted in a double layer closure of the ear canal. In case of previous meatoplasty, closure of the ear canal required a larger periosteal flap and delicate preparation of the thin ear canal skin. The medial skin of the bony external ear canal was dissected circumferentially and removed, allowing a proper canaloplasty to have sufficient visual control before precise removal of the tympanic membrane with the tympanic annulus, ossicles (malleus and incus, if still present) and the remnants of the ear canal skin.

Next, removal of all accessible air cell tracts with skeletonisation of the middle and posterior fossa dura, sigmoid sinus, facial nerve and jugular bulb was performed, followed by meticulous removal of the mucous membrane in the middle ear spaces. This step often required thorough removal of scarred soft tissue after previous surgeries, dissection of cholesterol granulomas filling many small, pneumatised cells in the retrofacial, perilabyrinthine and supralabyrinthine spaces. The stapes suprastructure was either missing or necessitated careful removal to allow cleaning of diseased mucosa from the mobile footplate. The Eustachian tube ostium was closed with bone wax, and the tensor tympani muscle elevated from its canal.

After meticulous irrigation of the operative field, the bony bed for the implant was created and a cochleostomy or round window membrane incision and electrode insertion was performed. Finally, the cavity was obliterated with abdominal fat and in situation (A) with the musculoperiosteal flap from the mastoid and occipital bone or in situation (B) using the temporalis muscle rotated inferiorly over the cavity. The skin incision was closed in two layers without suction drains. A circumferential head band with slight pressure was applied for 1–2 days [5, 7, 10].

In a staged procedure the first stage consisted of an SP as described above, however, without drilling the implant bed and without implantation. The staged implantation was scheduled at least 6 months later. In three patients a non-EPI DW MRI was performed, to rule out cholesteatoma prior to implantation. The retroauricular incision was re-opened (without the superior prolongation), the muscle flap was incised and the fat underneath was partially removed or elevated anteriorly, identifying the bony landmarks. The mastoid portion of the facial nerve and the promontory were identified and either a cochleostomy or a round window approach was chosen. The implant bed was drilled underneath the muscle flap and following the implantation, additional fat was harvested and used to fill the cavity again. No drain was placed and a pressure bandage applied for 1–2 days.

## Results

In 14 patients implantation was performed in a single stage and in 5 cases in two stages. Follow-up ranged from 8 months to 10 years and in 12 patients the follow-up was longer than 2 years.

In two staged cases no difficulties were found in round window localisation. In one case the scala tympani was ossified and successful full insertion was done via the scala vestibuli. In seven patients subtotal petrosectomy was performed after previous open cavity surgery and in three patients cholesteatoma was diagnosed before surgery. Despite enlarged external ear canals after previous meatoplasty in open cavity patients, meticulous ear canal skin closure was achievable in all cases without any delay in would healing. Cosmetically the plane of closure became more lateral, but was even less noticeable than the large entrance before.

Two stages were applied mainly in patients with discharging ears in whom therapy based on bacterial culture was not efficient. In those patients second stage surgery with cochlear implant insertion was performed after non-EP DW MR (non-echoplanar diffusion weighted magnetic resonance) excluded the presence of residual cholesteatoma at least 6 months after first stage subtotal petrosectomy. All the patients use their implants and there were no major nor minor complications. In one patient a postoperative CT showed aeration in the region of Eustachian tube ostium. No signs of infection were noted otherwise and the patient did not require any additional treatment. He was advised not to blow his nose forcefully during upper respiratory infections and to use antibiotics in case of upper airway infection with suspected bacterial origin. Detailed information with patients’ clinical data is presented in Table 1. All patients received perioperative antibiotics (most commonly second-generation cephalosporin or other depending on preoperative bacterial culture).

**Table 1:** Clinical data of patients with chronic otitis media requiring cochlear implant treated with the use of subtotal petrosectomy

No.	Initials, age, sex, side	Open cavity	Other previous surgery	Cholesteatoma	Otorrhea	Other pathology/difficulties	Two stages	Period between stages	Length of follow-up
1	(BM), 49, F, left					Temporal bone malformation (low middle fossa dura, anterior sigmoid sinus), retinitis pigmentosa			10 years
2	(BM), 58, F, right		Yes			Temporal bone malformation, retinitis pigmentosa			4 years
3	(BV), 65, F, right					Morbus Widal			1 year
4	(HP), 57, F, left		Yes			Middle ear atelectasis			4 years
5	(HP), 59, F, right	Yes							6 months
6	(IK), 56, M, right	Yes				Severe diabetes mellitus			2 years
7	(LA), 82, F, Left		Yes		Yes				1 year
8	(SR), 66, M, right		Yes	Yes					6 years
9	(VS), 31, F, left	Yes							2 years
10	(WE), 60, F, Left					Osteoradionecrosis after cobalt irradiation			1 year
11	(OT), 54, F, Left		Yes						7 years
12	(ST), 51, F, right	Yes							7 years
13	(HL), 48, F, right				Yes	Massive granulation in the mastoid and middle ear	Yes	6 months	18 months
14	(ZM), 65, F, left	Yes			Yes	Ossification of scala tympani, full insertion via scala vestibuli	Yes	6 months	4 years
15	(CB), 75, F, Left	Yes							2 years
16	(DA), 64, M, right			Yes	Yes	Otorrhea	Yes	11 months	8 months
17	(MS), 39, F, left	Yes		Yes		Large previous meatoplasty			3 years
18	(DP), 49, F, right				Yes	Wegener’s granulomatosis, exposed facial nerve in a previous subtotal petrosectomy	Yes	6 months	2 years
19	(JP), 12, M, right				Yes	Temporal bone malformations	Yes	6 months	1 year

## Discussion

The cochlear implant surgeon facing the decision to implant a patient with chronic otitis media (with or without cholesteatoma) or a patient with failed previous tympanomastoid surgeries has to consider the following realities:

- Hearing preservation surgery with a subsequent option of electroacoustic stimulation is very unlikely, since most of these patients have either total deafness or at least severe mixed hearing loss and sound conduction through the infected or previously operated middle ear by acoustic stimulation is not attempted.
- Eradication of the disease, avoidance of recurrence and prevention of implant infection or extrusion are primary goals.
- In case of additional temporal bone or inner ear malformations, prevention of cerebrospinal fluid leak or late meningitis is an additional objective.

There have been several techniques proposed in the literature to manage patients with chronic otitis media requiring a cochlear implant. They can be divided into three groups:

(A) “Covering techniques” to avoid electrode extrusions by wrapping the electrode cable into dense patient’s own tissues: Schlondorf et al. [11] used full thickness postauricular skin and soft tissue flap to cover the electrode in the mastoid cavity. Manrique et al. [12] suggests using tragal cartilage and fascia to cover and hold the electrode in the cavity. Others suggest reconstructing of the tympanic membrane and posterior ear canal wall [13]. Kojima et al. [14] used a canal wall reconstruction technique with mastoid obliteration in 2 patients with open cavities. However, Olgun et al. [15] reported 37 cases with COM and existing open cavities or middle ears converted to open cavities prior or at the time of cochlear implantations. In 7 (19 %) of them the electrode cable disrupted the epithelial lining of the cavity and reimplantation was necessary. El-Kashlan et al. [16] recommended the closure of external ear canal and leaving intact the pneumatised mastoid that would enable later serial CT follow-up. Roehm and Gantz [17] presented a case of chronic otitis media that developed after cochlear implantation and resulted in the need of explantation. They also reviewed the literature from 1995 to 2004 for papers describing complications of cochlear implantations in COM patients. In 14 from 100 patients a reoperation became necessary and in 7 cases the implant had to be removed due to complications. None of those seven patients was operated with our SP technique and in fact two of them required SP as a treatment of previous complications.

All those techniques carry the lifetime risk of electrode exposure and extrusion with subsequent need to remove the implant. They also do not guarantee a solution against recurrent or residual disease and in case of inner ear malformation a CSF leak would pose a serious problem. We therefore strongly discourage from using these “covering” techniques.

(B) *“*Bypass techniques*”* using a surgical approach for electrode insertions away from the diseased middle ear and mastoid: Kojima et al. [14] used a transcanal approach drilling the grove in the posterior canal wall in patients with adhesive otitis to avoid drilling in a previously infected and scarred mastoid. Olgun et al. [15] advocated the use of a subfacial approach in those cases with previous modified radical cavities and reported stable results in 13 patients with a follow-up from 1 to 5 years. Colletti et al. [18] reported a method of electrode insertion via the middle fossa approach in cases with chronic otitis media. This technique requires a craniotomy, carries higher risk of facial nerve injury and leaves the middle ear disease unsolved. In patients with open cavities the risk of continuing recurrent discharge from the ear remains. Again, we strongly discourage from using any of these “bypass techniques”, since the underlying problem is not solved.

(C) “Subtotal petrosectomy” technique to eliminate the chronic middle ear and mastoid disease and implantation of electrodes into a clean surgical field: Fisch and Mattox [5] described the detailed technique of SP already in 1988 and 10 years later he introduced the concept for patients in need of a cochlear implant. Among the first five patients were patients with temporal bone and inner ear malformations, meningocele and chronic otitis media [7]. It took another 10 years until cochlear implant surgeons became familiar with this concept and it is only now, that we can refer to various centres reporting their experience in the ENT literature. The Antwerp group summarized 29 patients with severe chronic otitis media resistant to medical or previous surgical treatments who underwent an SP. None of the patients had recurrent otorrhea, one had a residual cholesteatoma and one patient out of five who received a CI in a single-stage procedure revealed a severe complication: he had two consecutive flap failures with wound breakdown 6 and 5 months after revision surgeries and finally required explantation and re-implantation [19].

In the group of 32 patients operated by means of SP combined with cochlear implantation at Piacenza reported by Free et al. [6], 4 had chronic suppurative otitis media and 13 patients had previous canal wall down surgeries (one with an electrode extrusion through the retroauricular skin of the modified open cavity). One patient (3 %) in the SP group developed a retroauricular wound infection with granulation tissue requiring reoperation and repositioning of electrode array. Our group of 19 patients had a follow-up until 10 years with no early or late complications. Postelmanns et al. [20] described a group of 13 patients with COM out of 156 (8.3 %) patients that received cochlear implants in Maastricht. In eight patients in this group SP was performed without any complications. In the remaining five patients one major complication occurred requiring explantation of the CI. The patient had simple myringoplasty for a dry perforation and developed skin infection leading to reoperation and finally to the explantation of the device. They advocate staged procedure—first SP and after 3–6 months cochlear implantation—in any case of active disease, including any case with cholesteatoma.

More recently, Bernardeschi et al. [21] reported 24 patients with chronic otitis media treated with single-stage cochlear implantation and subtotal petrosectomy from the group of 30 cases treated with this technique. They strongly advocate one-stage surgery in all patients with prolonged antibiotic therapy in case of positive bacterial culture. They reported no complications except two abdominal hematomas nor cholesteatoma recurrence; however, the follow-up in six of the reported cases was only 3 months.

We strongly favour the SP technique for patients with chronic otitis media, previous modified radical cavities or failed tympanomastoid surgeries who require a cochlear implant procedure. The SP allows maximum exposure of the temporal bone, reveals a high chance of radical removal of any disease (minimal risk of residual disease) and avoids any recurrence of the disease (e.g. further middle ear atelectasis is not possible since the drum was removed), and limits the risk of meningitis in cases of additional inner ear malformations. Although we have not specifically addressed the hearing outcome, generally speaking, the functional outcome was similar to cochlear implants in healthy middle ears, as observed also in the Antwerp and Piacenza group of patients [6, 20].

The decision in these cases is therefore not, if there are other alternative techniques to the SP, but whether to stage the procedures or to perform a single-stage implantation.

In 14 (74 %) patients from our group a single-stage surgery was performed. Obvious advantage of this strategy is that patient has just one surgery and in 3–4 weeks the cochlear implant is activated. Because of the risk of biofilm formation on the implant, in patients with active discharge from the ear with multiresistant bacterias, extensive cholesteatoma or in previously irradiated temporal bones, staged surgery may be necessary. Disadvantage of single-stage surgery is that imaging with diffusion—weighted MRI is not easily possible anymore after cochlear implant placement (or the magnet needs to be removed), making an early diagnosis of residual (not recurrent) cholesteatoma impossible. In three of our patients cholesteatoma was present and in two of them single-stage surgery was performed. In one case the procedure was staged and second stage performed after non-EP DW MR excluded residual disease. We suggest performing imaging in all two-stage cases 6 months or longer after the first stage. The period between stages should be at least 6 months as non-EP DW MR imaging technique is able to detect pearls of cholesteatoma larger than 2–3 mm depending on the study protocol and class of the MR equipment [19, 22]. In case of a positive finding, this small residual perl can be extracted at the same time as the staged CI implantation takes place. As we are aware that small pearls or a flat residual cholesteatoma matrix may not be detectable at 6 months postoperatively, we suggest that an MRI is optional and not mandatory. If the period is too short small residual pearls enlarging in the obliterated cavity may be missed radiologically and even during second stage surgery, since the fat pad is not completely removed at the second stage. Therefore, careful surgical inspection of the cavity should be performed during the second stage. In patients operated with a two-stage technique the fat obliterating the cavity is partially removed allowing the identification of the previous bony margins of the cavity, the promontory, round and oval window niches. After cochlear implant insertion the cavity is further filled with freshly harvested subcutaneous fat. In all our patients perioperative antibiotic coverage was used in both stages to further reduce the risk of implant infections.

Recently Vincenti et al. [23] reported long-term results of 12 patients after subtotal petrosectomy and single- or two-stage cochlear implantation in patients after open cavity surgery. They reported one patient with residual cholesteatoma and one with wound breakdown at the external meatus. They also advocate follow-up HRCT about 1 year after surgery and further imaging depending on clinical symptoms. The use of subtotal petrosectomy and implantation of middle ear implants has also been reported [24].

**Fig. 3:**
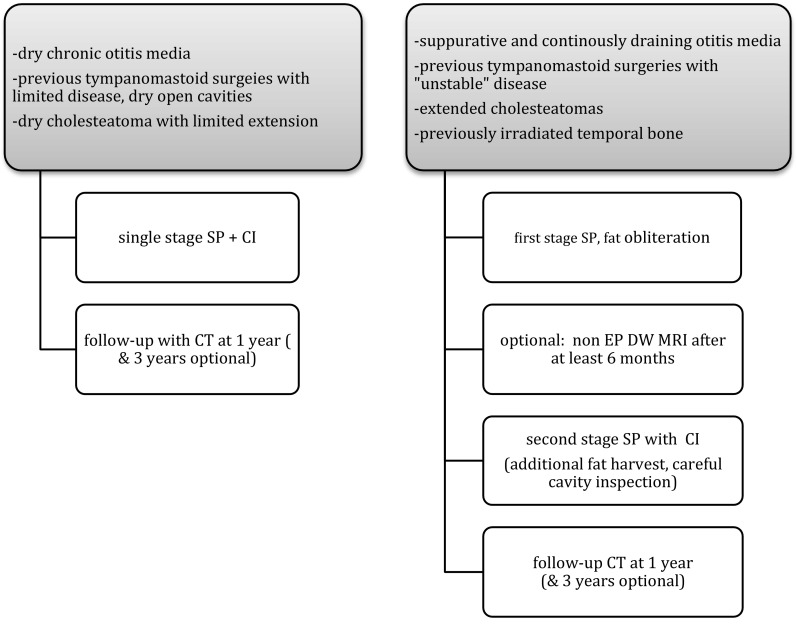
Treatment algorithm in patients with chronic otitis media requiring cochlear implant

Looking at our own series and experience and reviewing the literature, we suggest the following treatment algorithm (Fig. 3).

Main indications are patients with chronic otitis media with or without previous surgeries, CI candidates with inner ear or temporal bone malformations limiting the surgical exposure (posterior tympanotomy) or harbouring the risk of CSF leak and meningitis including also transverse temporal bone fractures. The surgeon has to decide upon a primary or staged implantation. A follow-up CT scan (or Cone beam CT) performed 1 year after cochlear implant insertion confirms the air tight closure of the Eustachian tube (no air in the protympanum), identifies the position of the electrodes within the cochlea and in case of doubt of residual disease can be used as a baseline scan for comparison 3–5 years later. Pathologies within the fat pad may be identified due to differences in tissue density in Hounsfield unit scale. Furthermore, serial CT examination may show growth of the lesion within the obliterated cavity suggesting residual cholesteatoma [6].

The only contraindication would involve a CI-candidate with an attempt for electroacoustic amplification. However—as mentioned above—this is very unlikely due to the disease process both in the middle ear and its previous effects on the inner ear.

## Conclusions

- The use of subtotal petrosectomy with cochlear implants is a safe and efficient technique when strict surgical steps and rules are applied.
- The flap design is different between primary surgeries and revision surgeries.
- Closure of the external ear canal after previous meatoplasty can be challenging and extreme care dissecting the skin flaps is required.
- In patients with extensive cholesteatoma, active discharge from the ear with resistant bacteria or an “unstable” situation following previous irradiation, the procedure should be staged.
- 25 years after its introduction, the SP has gained widespread acceptance in temporal bone surgery.

## Electronic supplementary material

Below is the link to the electronic supplementary material.

Supplementary material 1 (MPG 189738 kb)
